# Microbial diversity in the vaginal microbiota and its link to pregnancy outcomes

**DOI:** 10.1038/s41598-023-36126-z

**Published:** 2023-06-04

**Authors:** Agnes Baud, Kenzo-Hugo Hillion, Céline Plainvert, Véronique Tessier, Asmaa Tazi, Laurent Mandelbrot, Claire Poyart, Sean P. Kennedy

**Affiliations:** 1 Institut Pasteur, Université Paris Cité, Département de biologie computationnelle, F-75015 Paris, France; 2 AP-HP Centre-Université Paris Cité, FHU PREMA, Centre national de référence des streptocoques, Paris, France; 3 FHU PREMA, AP-HP DRCI, Paris, France; 4 Service de gynécologie-obstétrique, Hôpital Louis Mourier, AP-HP, Université de PARIS, IAME INSERM U1137, Paris, France; 5 Université de Paris, INSERM, Institut Cochin 1016, Paris, France

**Keywords:** Clinical microbiology, Metagenomics, Microbiome, Preterm birth, Risk factors, Predictive markers

## Abstract

The vaginal microbiota refers to the microorganisms that reside in the vagina. These microorganisms contribute significantly to a woman’s reproductive and general health. A healthy vaginal microbiota is typically a low-diversity environment with a predominance of lactic acid-producing *Lactobacillus* species. Factors such as antibiotic use, sexual activity, and hormonal changes can disrupt the balance of the vaginal microbiota, leading to conditions such as bacterial vaginosis. The composition of the vaginal microbiota changes and takes on added importance during pregnancy, serving as a barrier against infection for both mother and fetus. Despite the importance of the microorganisms that colonize the vagina, details of how changes in composition and diversity can impact pregnancy outcomes is poorly understood. This is especially true for woman with a high prevalence of *Gardnerella vaginalis*. Here we report on a diverse cohort of 749 women, enrolled in the InSPIRe cohort, during their final trimester of pregnancy. We show that *Lactobacilli*, including *L. crispatus* are important in maintaining low diversity, and that depletion in this critical community is linked with preterm delivery. We further demonstrate that it is overall diversity of the vaginal microbiota, not specific species, which provides the best indicator of risk.

## Introduction

As a whole, human microbiota comprises the ~ 10^11^ microorganisms, predominantly bacteria, living on and within each individual^1^. The microorganisms present in the vagina, collectively called the vaginal microbial community (VMC), is specific in both its composition and its function in women. The human intestinal tract harbors the majority of the microbiota in terms of both diversity and numbers while the VMC is distinct in composition and generally presents a much lower diversity of microorganisms^2^. The VMC plays a critical role in the health of women in general and in reproductive-aged women specifically. The composition of the vaginal consortium goes through multiple distinct phases starting shortly after birth, through until menopause^3^. Changes to the vaginal environment of reproductive-age women results from ovarian steroid hormone cycling, with estrogen linked with increased glycogen availability^4,5^. These effects are further amplified during pregnancy as estrogen levels increase during gestation and the VMC during pregnancy has been shown to be significantly different from that of non-pregnant women of child-bearing age^6^. During pregnancy, the VMC community interacts with the host to provide a protective barrier against potential pathogens for the developing fetus. The breakdown of free glycogen by α-amylase provides a source of rich energy source that can be converted to lactic acid thereby lowing the overall pH of the environment^7–9^.

The production of lactic acid is assured almost entirely by species from the *Lactobacillus* genus. As first described by Döderlein^10^, and further elaborated by Rogosa et al.^11^, culturing, microscopy, and molecular methods have shown the VMC is dominated by species of the *Lactobacillus* genus^12^. By maintaining an acidic pH below 4.5, *Lactobacillus* spp. limit colonization by other taxa including potential pathogens. Modern molecular techniques including high-throughput sequencing evinced a distinct structure in the VMC centered around *Lactobacillus* species. Ravel et al*.* used 16 s rDNA sequencing and analysis to define four major community sequence types (CSTs) dominated by *Lactobacillus crispatus* (CST I)*, L. gasseri* (CST II)*, L. iners* (CST III), and *L. jensenii* (CST V). A fifth CST was typified by a lower proportion of lactic acid producing bacteria and a relatively higher proportion of anaerobic organisms including the species *Gardnerella vaginalis* (CST IV)^13^. *Gardnerella vaginalis* prevalence has long been considered to be a dysbiotic CST often associated with bacterial vaginosis (BV) and higher risks to health.

Bacterial vaginosis is a common form of dysbiosis affecting reproductive aged women. BV is typified by reduced *Lactobacillus* abundance in the VMC, resulting in an increased pH > 4.5 and a commensurate increase in diversity^14^. BV is most often associated with an abundance of *Gardnerella vaginalis* in CST IV, often accompanied with an increase in the number of anaerobes, *Bacteroides* and *Prevotella,* and often a range of potential pathogens including *Ureaplasma* and Gram-positive *Mobiluncus* and *Megasphaera*^15–17^. Although early studies identified *Gardnerella vaginalis* as the primary cause of BV, it is now recognized that the vaginal bacterial community varies widely in different regions and across different genetic backgrounds^18^. Indeed, the ethnic background of women is an important criterion in assessing clinical phenotypes and risk profiles. A report of European pregnancies indicated that only 2% of women had a *Gardnerella vaginalis* type IV CST, while a similar study of African women reported that nearly 50% of subjects had this CST^19,20^. Whole genome shotgun metagenomics can be applied to provide species and strain-level resolution in order to better comprehend these population differences and their effect of the health of both mother and infant. A comprehensive catalog of genes and species derived from whole genome metagenomics of the VMC revealed a rich environment with 300+ species and nearly a million predicted genes^21^. Our improving appreciation of VMC complexity, coupled with its interaction with the host during pregnancy, open new avenues to elucidating its role in unfavorable pregnancy outcomes such as premature birth.

Preterm birth refers to the birth of a baby before 37 weeks gestation. Preterm births represent 11.1% of global livebirths and are a significant cause of infant morbidity and mortality^22^. Nearly one million deaths among children under the age of five can be directly traced to complications of preterm delivery^23^. Preterm babies often require special medical care in neonatal intensive care units, and may face challenges such as breathing difficulties, feeding problems, and developmental delays. Outside of the approximately 20% of preterm deliveries which are medically induced, our understanding of the etiology of preterm birth remains imprecise. Infections appear to account for 30–40% of spontaneous preterm births with a combination of other factors, including multiple pregnancies, racial origin, age, smoking, high or low maternal BMI and others, contributing to the difficulty in assessing risk^23^.

As stated above, the protective role of the vaginal microbiota against infection is reinforced during pregnancy as hormonal changes contribute to the increased dominance of *Lactobacilli*^24^. In addition to its protective role, the VMC is also likely responsible for the first important microorganism colonization event of the newborn which occurs during vaginal delivery; the passage through the birth canal imparting some of first constituents of the newborn oral and intestinal microbiota^25,26^. It has been largely assumed that, in contrast to robust diversity in the intestinal microbiota, low diversity and dominance by a single *Lactobacillus* species is the preferred state during pregnancy. Nevertheless, it has been recently reported that the structure of the vaginal microbiota in late-term pregnancy changes, increasing in diversity, to potentially aid in this initial colonization step^27^. Another study demonstrated, in a mouse model, that the composition of the human VMC has a detectable effect of both the offspring microbiota and health^28^. As several VMC species, at delivery, are also early colonizers of the newborn microbiota, more attention has been paid to this community, its composition throughout pregnancy and its association with perinatal infections and preterm birth.

Whether due to BV or other type of disruption to the healthy vaginal microbiota, there is intense interest in understanding how the risk of unfavorable pregnancy outcomes are linked with variations in the composition of the VMC^29^. The risk of pre-term delivery and premature rupture of the amniotic membrane before delivery have been associated with the presence of specific organisms. Although much attention has been paid to shifting bacterial population and a reduced proportion of *Lactobacillus* as the cause for various risks during pregnancy, the presence of viruses and fungi, specifically *Candida albicans*, has also been observed with pre-term delivery risks^30,31^. Here we investigate the role of the vaginal microbiota, its diversity, and the prevalence of different community types in relation to pregnancy outcome.

## Materials and methods

### Ethics declaration

The reported study (RCB # 2017-A02755-48) was reviewed by the French ethics committee, “Comité de Protection des Personnes Nord-Ouest III” (Caen, France), who granted approval on November 4th, 2017 (CPP dossier: 2017-74). All individuals enrolled in the InSPIRe cohort provided signed informed consent that included authorization for the collection of medical information and biological samples. The present work includes only the non-human fraction of DNA extracted from vaginal swabs from expectant mothers. Clinical metadata of patients was collected in a secured electronic database. Exports from the database used for analysis were anonymized. Processed vaginal swabs were labeled with corresponding anonymous inclusion numbers.

### DNA extraction

Vaginal swabs collected with E-swab collect tubes and containing 1 ml of AIMES conservation solution were immediately frozen and stored at – 80 °C. Samples were shipped on dry ice to Eurofins (Aarhus, DK) for DNA extraction. Briefly, samples were thawed on ice and vortexed to obtain a homogenous solution. For lysis, 750 µl of sample was centrifuged and resulting the pellet resuspended in 540 µl of buffer ATL and 60 µl of Proteinase K. Two steel-beads were added, and the sample shaken in a Tissuelyser instrument at 2 × 2 min at 20 Hz followed by incubation at 56 °C for 15 min. 500 µl of the lysates was transferred to Sarstedt tubes and inserted in to the QIAsymphony (Qiagen: Düsseldorf, Germany) extraction robot. Automated extraction of DNA from 750 µl swab medium was performed using the QIAsymphony DSP Virus/Pathogen Kit and the Complex400_OBL protocol. DNA was eluted in 60 µl Qiagen AVE buffer into 96-well plates containing 95 samples and one negative control.

### Whole-genome metagenomic sequencing

Metagenomic libraries were prepared using Illumina (San Diego, CA) TruSeq kits. Extracted DNA was fragmented to an average size of 500 bp using a Covaris sonicator. Ends were repaired prior to ligation of sequencing and barcoding primers. Following PCR amplification of the libraries, insert size was confirmed with an Agilent 2100 and qPCR was performed to ensure an effective library concentration of > 3 nM. Libraries were sequencing on a NovaSeq6000 instrument. An average of 6 Gb of high-quality data was targeted for each sample, corresponding to 2.0 × 10^7^ paired-ends reads (2 × 150 bp).

### Customized database and taxonomic assignment

We performed taxonomic profiling of 600 + human samples (oral, fecal, breast milk and vaginal) using MetaPhlAn3^32^. Identified genera were used to filter the ‘maxikraken2_1903_140GB’ Kraken2^33^database; archaea, bacterial, fungi and protozoa species from these genera were retained. Assemblies for species not present in Kraken2 libraries were obtained from NCBI. When multiple assemblies were available, we prioritize the RefSeq tag "reference genome" over "representative genome" over "na." Remaining "excluded_from_refseq" species were used if labeled "genus undefined", "derived from environmental source", "derived from metagenome", "derived from single cell" or "sequence duplications." For assemblies, "Complete Genome" was preferred over "Chromosome", then "Scaffold" and lastly "Contig". The most recent assembly was selected for any redundant datasets. 13 species could not be found using this method; all but *Coprobacter secundus* and *Flavobacterium thermophilum* had at least one other representative of their genus already present. The Kraken2 script kraken2-build was used to build the customized database, CustomDB, from the resulting FASTA sequences, comprising 1302 species from 225 genera. Kraken2 was then used, with CustomDB, to assign taxonomy to sample reads.

Phylogenetic tree for taxonomic diversity. We identified 16S sequences from either SILVA^34^ or NCBI nucleotide databases for 1215 out of 1302 species present in CustomDB. Sequences were aligned using the Multiple Sequence Alignment tool, MAFFT (Multiple Alignment using Fast Fourier Transform)^35^, with the option –auto. A phylogenetic tree was generated with RAxML (Randomized Accelerated Maximum Likelihood)^36^ using a random seed (-p 1234), under the General Time Reversible model of nucleotide substitution and the Gamma model of rate heterogeneity (-m GTRGAMMA). This model includes different rates of substitution for each pair of nucleotides and across the sequence, as well as different frequencies of occurrence of nucleotides. We retained the tree with the best likelihood upon 100 bootstraps (-N 100). The remaining 87 species were added in three steps: (1) For each missing species, the Most Recent Common Ancestor (MRCA) of species from the same genus, present in the tree, was calculated using the python library dendropy. If no common genus was found, the family level was used. (2) The mean distance of all the species of the same genus (or family) to the MRCA was calculated; (3) Missing species were added at the MRCA node with a length equal to the calculated mean distance.

### Diversities computation

Alpha-diversity (intra-sample richness) and beta-diversity (inter-sample diversity) were each computed using two methods which differed in their inclusion, or not, of phylogenetic distances. For the alpha-diversity, we computed the Shannon diversity index^37^ of all 749 samples, and the Faith’s phylogenetic distance^38^. For the beta-diversity, we computed the Bray–Curtis dissimilarity^39^, as well as the weighted UniFrac^40^. The alpha-diversities were calculated based upon the number of taxa identified, with Kraken2 using CustomDB, from samples that had been downsized to 10 k reads. Beta-diversities were computed based upon taxa identified, with Kraken2 using CustomDB, with all reads after by DESeq2-type geometric mean normalization^41,42^. We evaluated the significance of the differences in diversities with a Mann–Whitney-U statistical test and corrected the p-values by False Discovery Rate correction with the Benjamini–Hochberg method.

### Principle Component Analysis (PCoA)

PCoA was computed in Python (ver. 3.7) using the stats-ordination module of scikit-bio (http://scikit-bio.org, ver. 0.5.6). The pcoa function was used to calculate plot coordinates, taking a Bray–Curtis dissimilarity matrix as input. The pcoa_biplot function was used to extract component (taxa) contribution vectors. Bray–Curtis dissimilarity was calculated using the diversity.beta-diversity module of scikit-bio. Intermediate data including sample coordinates for each principal component, and vector for each taxon component, were collected as Pandas (https://pandas.pydata.org, ver. 1.0.1) data frames and visualized using Plotly (https://plotly.com, ver. 5.3.1).

### Random forest modeling and prediction

Machine learning analysis of the data was implemented by training a random forest (RF) classifier. Features used to train the classifier were normalized counts of species for each sample. We used the RandomForestClassifier in the Python package sklearn (https://scikit-learn.org, vers. 0.24.3) for model training and sample classification. Results were tested and interpreted using the model_selection and metrics functions of sklearn. The matplotlib package (https://matplotlib.org, ver. 3.4.2) was used to plot the results. Training of the RF classifier was run with 10^5^ estimators. In order to obtain features with the strongest signal, the classifier was trained with features present in at least 25% of samples and with a minimum mean of 50 counts. Recursive Factor Removal (RFR) was performed by iteratively removing the factor with the weakest contribution to the RF model and retraining. The performance of each model was assessed, and locally weighted smoothing (LOWESS) applied to determine the optimal number of features. The final model was trained on the highest performing features and tested by generating a receiver operating characteristic (ROC) curve for three independent train/test sample sets.

## Results

### A prospective study of pregnant women

We established a prospective cohort of 2313 pregnant women with the goal of studying the vaginal microbiota, its community composition and the presence of pathogenic bacteria in the peripartum period. Inclusions and data collection were performed in accordance with applicable laws and ethical standards. Ethical approval for the study was obtained in November of 2017. Patients were enrolled, and samples collected, at three French hospitals in the Paris region (AP-HP): Hôpital Bichat Claude-Bernard, Hôpital Louis-Mourier and Hôpital Port-Royal. Informed consent was obtained prior to inclusion. In order to be included in the study, individuals had to have passed their 22nd week of gestation and be at least 18 years old. This work reports on 749 mothers whose pregnancies stared between 2018 and 2020, where vaginal swabs were collected through June of 2021 and whose clinical data records had been completed and validated.

**Figure 1:**
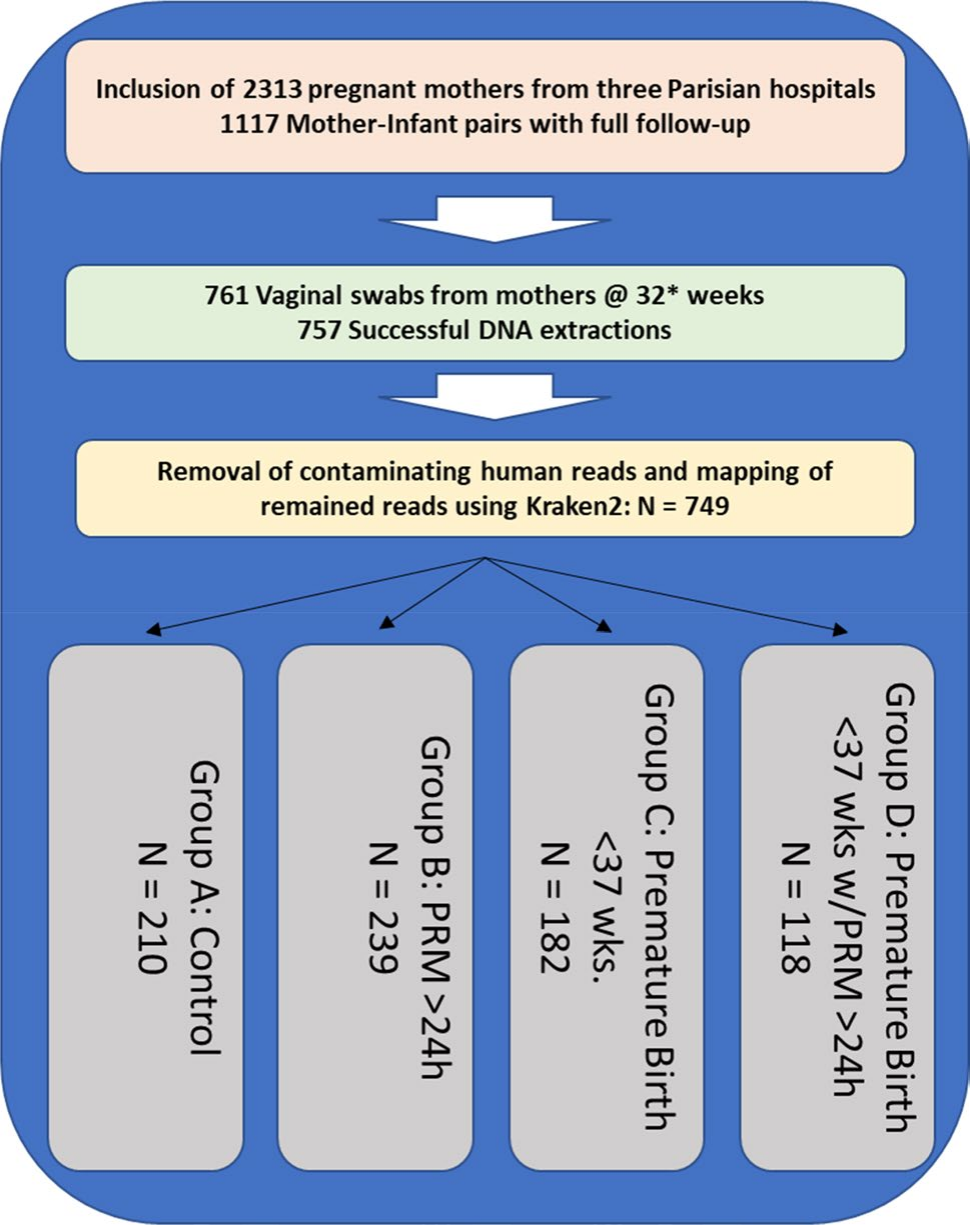
Cohort Composition and distribution of samples. 2313 pregnant mothers were recruited and consented to participate in the observational cohort. Current follow-up of mother-infant pairs with full clinical records equals 1117 completed dossiers. A total of 761 samples were selected for analysis. Four samples were excluded due to poor DNA extraction yield and a further eight samples did not yield sufficient sequence data to be included in the analysis. The distribution of samples across groups A, B, C and D is shown.

The cohort, summarized in Fig. 1, was organized into four groups: (A) The control group of women who underwent standard-of-care screening at 32 weeks of pregnancy and who displayed no clinical risk of premature delivery or premature membrane rupture, (B) Full-term pregnancies in which there was a premature rupture of the membrane > 24 h before childbirth, (C) Premature births defined as labor before 37 weeks of gestation and (D) Premature deliveries that also experienced membrane rupture > 24 h before childbirth. Vaginal swabs were collected from the mother at the time of delivery.

Categorical electronic patient records were used to collect both anthropomorphic as well as clinical data for individuals in the cohort, with the resulting information stored in a secure database. The health status of mothers was recorded using a set of variables used to identify complication or risk factors such as gestational diabetes, smoking or alcohol use during pregnancy, or high BMI. Bacteriological culture was performed, plating vaginal swabs on several media and allowing for the detection of *Streptococcus agalactiae* (group B *Streptococcus,* GBS) and additional potential pathogens including *Escherichia coli, Klebsiella pneumonia, Proteus mirabilis*, among others. Antibiotic susceptibility or resistance to a number of routine antibiotics such as macrolides (e.g., erythromycin) and beta-lactams (e.g., methicillin or carbapenem) were measured using both plating and PCR methods. Data collection continued after delivery for mother-infant pairs in order to track immediate or delayed pathogen infections across the four cohort arms. The initial focus of our analyses has been on the microbial composition of samples and links with risk to pregnancy outcomes and infection. A full listing of the analyzed samples in this study as well as the clinical variables used for this study are provided as supplemental data (Supplemental Table S1).

### Dominant species influences pathogen presence in the vaginal microbiota

We analyzed maternal vaginal swabs from 761 of the completed mother-infant pairs. Sufficient DNA was successfully extracted from 757 samples. Samples were subsequently sequenced using Illumina paired ends (2 × 150 bp) technology. An average of 3.16 × 10^7^ (SD $\pm$ 1.4 × 10^7^) high-quality reads were generated for each sample. Although bacteria play an important role in the vaginal environment, their biomass in relation to human cell is low. Consistent with this fact, we found that human host reads made up a mean of 95% of the total data set. Human-mapping reads were removed prior to performing downstream analysis. Taxonomic identification of filtered reads was determined using Kraken2 to map with our customized vaginal microbiota database. An average of 1.06 × 10^6^ reads were mapped per sample. A total of 749 samples with matched patient records were retained for further analysis. Sequencing read identification and quantification for samples can be found in supplemental data (Supplemental Table S2).

**Figure 2:**
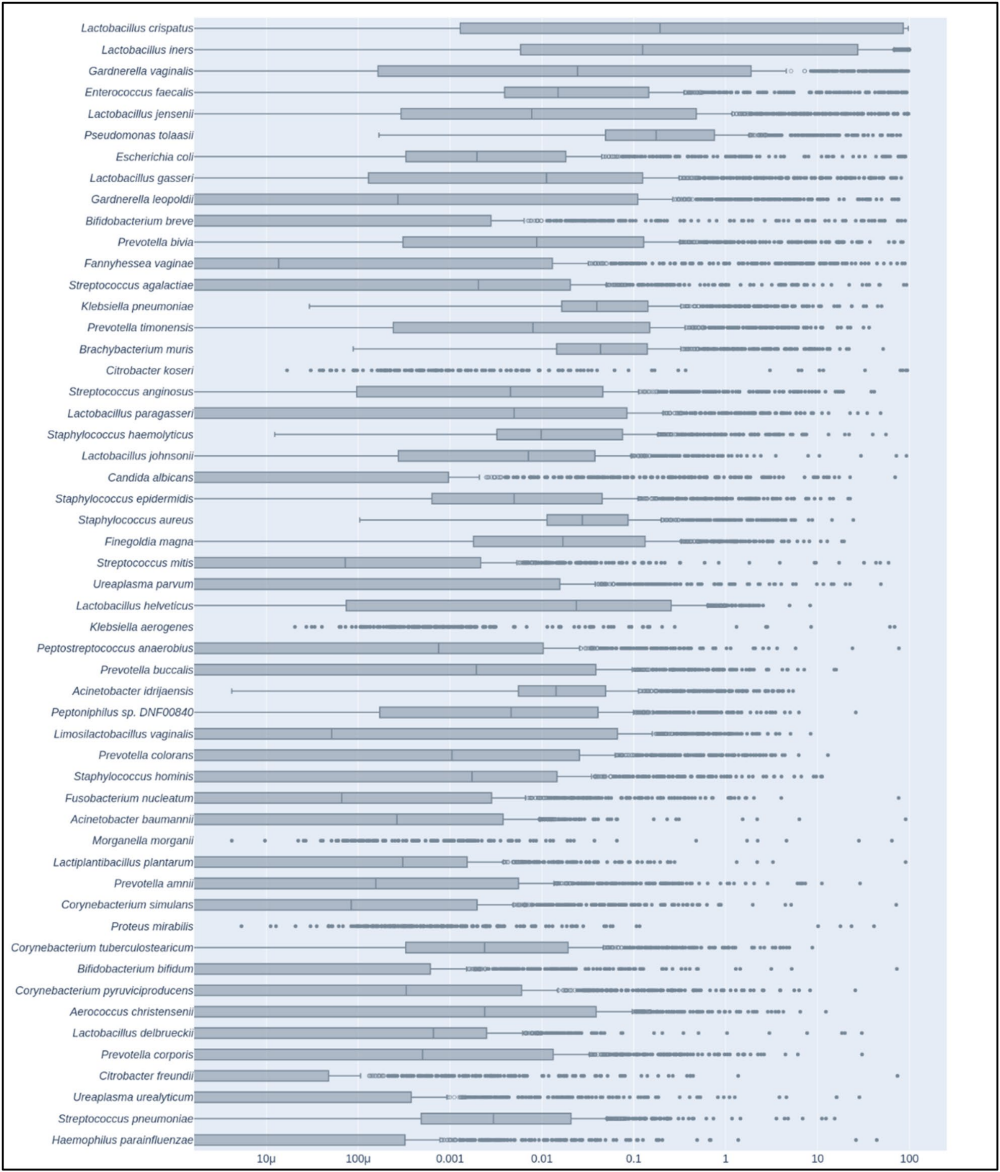
Relative abundance of the 53 most abundant microbial genomes among individuals of the cohort. Only species with a mean relative abundance across samples above 0.1% were retained for this graph. Boxes represent the interquartile range (IQR), delimited by the first and third quartiles (25th and 75th percentiles, respectively) and the line inside represents the median. Whiskers show the minimum and maximum values within 1.5 times IQR from the first and third quartiles respectively. Outliers (filled circles) are samples more than 3 times IQR below or above the first and third quartiles respectively. Suspected outliers (open circles) are samples between 1.5 and 3 times IQR below or above the first and third quartiles respectively.

We detected microbial communities of the vaginal microbiota mostly consistent with previously reported CSTs (community sequence types) (Fig. 2 and Supplemental Table S3). *Lactobacillus crispatus* (CST I) was the most abundant organism in our cohort, detected in 87% of samples and representing 30.18% of reads overall. *Lactobacillus iners* (CST III), 19.69% of total reads, was detected in 90.75% of samples and *Gardnerella vaginalis* (CST IV), 10.19% of total reads, was detected in 77.01% of samples. *Lactobacillus jensenii* (CST V), 3.42% of reads, and *L. gasseri* (CST II), 2.07% of reads, were also detected in > 75% of samples. Interestingly, we quantified several other bacterial species with relatively high total reads and high prevalence. *Enterococcus faecalis* was found in nearly 100% of samples and represented 3.88% of total reads. *Pseudomonas tolaasii*, 2.36% of reads, was detected in all samples while *Escherichia coli*, 2.36% of reads, was found in 83.75% samples. *Streptococcus agalactiae* (GBS), an important pathogen in both mothers and infants, was found in 74% of samples.

Our examination of the most prevalent organisms revealed that, aside from the expected dominance of *Lactobacillus* species in normal vaginal flora, a large proportion of the remaining prevalent species detected were either known or opportunistic pathogens. This result was exemplified by the detection of *Ureaplasma parvum* in 45% of samples and *U. urealyticum* in 31% of samples. These two species have been previously observed in pregnant women and are associated with asymptomatic carriage as well as pregnancy complications including premature birth^43^. *Candida albicans*, another organism potentially associated with an elevated risk of preterm birth^44^, was found to be present in 41% of samples. Examining their co-occurrence, we found *Candida* and *Ureaplasma* genera are found together in 20.08% of the total cohort. We did not initially identify any significant differences in co-occurrence that could be directly linked with cohort groups for full-term versus pre-term delivery. However, an examination of clinical parameters revealed that when grouped by hospitalization reason, samples from medically programmed Cesarean deliveries showed a 26.67% co-occurrence rate compared with 17.84% for individuals admitted for spontaneous vaginal deliveries. Furthermore, when we investigated co-occurrence across CSTs, we found that *L. jensenii*, *L. gasseri* and *L. crispatus* communities had the lowest rate of co-occurrence at 9.52%, 11.76% and 15.14%, respectively. G. vaginalis communities displayed the greatest rate of co-occurrence at 34.62% followed by *L. iners* at 24.68%. A chi-squared test indicated the observed percentages to be significantly linked with CSTs (p = 4.04 × 10^–4^).

### VMC Diversity and pregnancy outcomes

**Figure 3:**
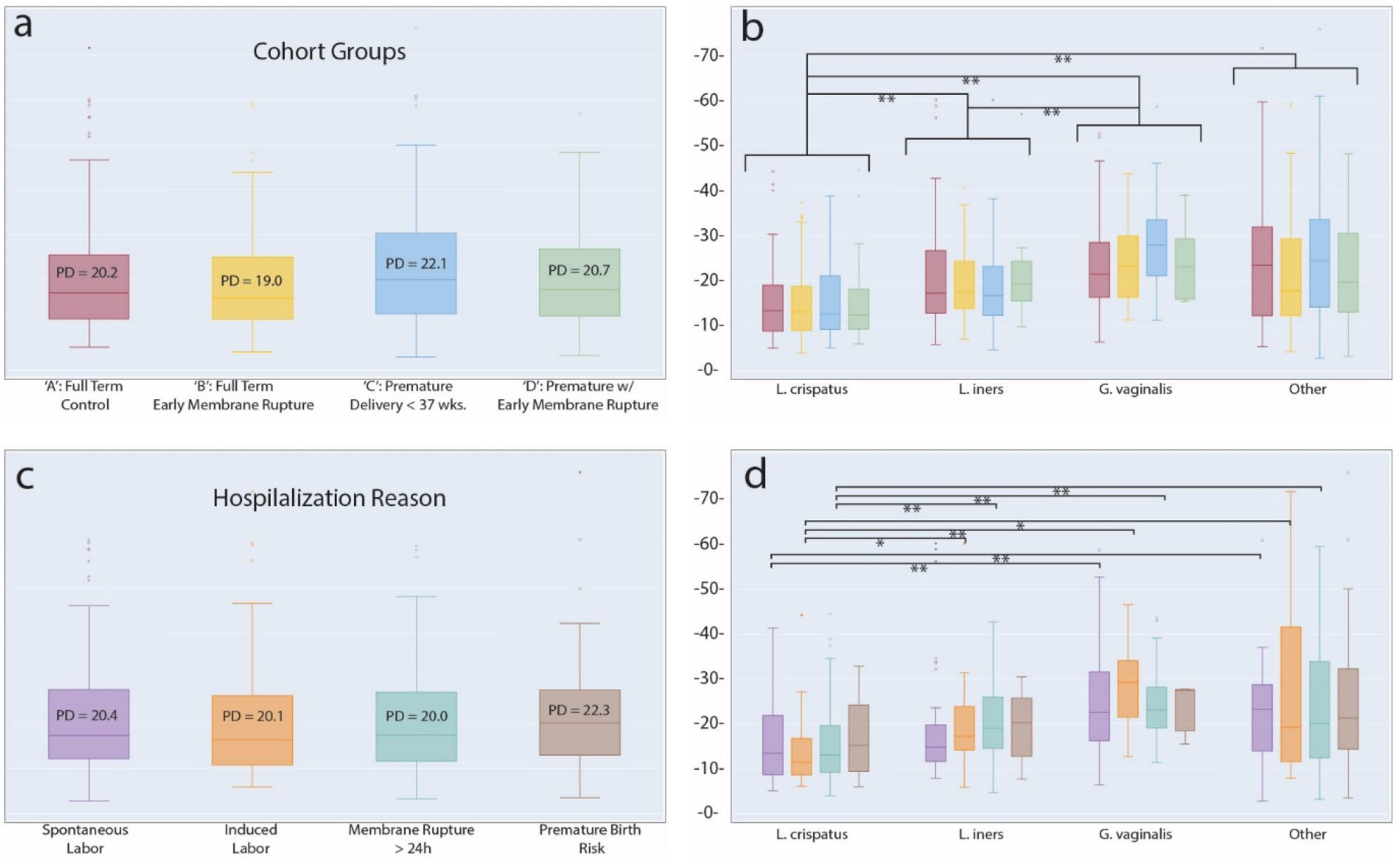
Faith’s Phylogenetic Diversity at the species level. (**a**) PD values for samples by ‘cohort group.’ No significant differences were found. (**b**) Samples grouped by dominant species, where a given species was most abundant in at least 100 samples. Each species group is subdivided by ‘cohort groups’ following the same color scheme as in ‘a’. Significant differences at the level of dominant species groupings are noted. (**c**) Samples grouped by ‘hospitalization reason’ for reasons with at least 40 samples. No significant differences were found. (**d**) Samples grouped by dominant species and subdivided by ‘hospitalization reason’ following the same color scheme as in ‘**c**’. *p < 0.05; **p < 0.01; False Discovery Rate, correction with Benjamini–Hochberg.

VMC diversity has been implicated in overall health and pregnancy outcomes with lower diversity community dominated by *Lactobacillus* species considered healthy with a lower risk of complications and infection^45^. We calculated the microbiota community α-diversity of vaginal samples using Faith’s phylogenetic diversity (PD) metric for the four groups of the cohort. Some differences were detectable in mean diversity between cohort groups (Control: PD = 20.2, PRM: PD = 19.0, Pre-term: PD = 22.1, Preterm with PRM: PD = 20.7), but these were not significant (Fig. 3a). A similar result was obtained when grouping samples according to hospitalization reason for mothers prior to delivery (Spontaneous Labor: PD = 20.4, Induced Labor: PD = 20.1, Membrane rupture: PD = 20.0, Risk of pre-term birth: PD = 22.3) (Fig. 3c). The elevated diversity in both the pre-term birth group as well as for the group hospitalized for an elevated risk of premature birth, while not significant, was nonetheless consistent with previous reports and was further investigated.

Having detected significant differences in VMC structure across CSTs, we followed up and stratified samples by their dominant bacterial species: *L. crispatus, L. iners,* and *G. vaginalis*. A fourth group, ‘other,’ included samples not dominated by one of these species*.* By using these bacterial groups, significant differences in diversity were uncovered, and were most striking, when comparing *L. crispatus* communities to the other three groupings (*L. crispatus* PD = 15.4, *L. iners* PD = 20.1, *G. vaginalis* PD = 25.1, ‘other’ PD = 23.7) **(**Fig. 3b). Independent Wilcoxon tests indicated significant differences between *L. crispatus* and *L. iners* samples (p = 7.02 × 10^–8^) and *L. crispatus* and *G. vaginalis* samples (p = 5.75 × 10^–17^). A weaker, but still significant difference existed when comparing *L. iners* and *G. vaginalis* (p = 9.72 × 10^–6^). Interestingly, there was no significant effect on diversity result when ‘other’ community types were calculated as either exclusive or inclusive of *Lactobacilli* from other common CSTs: *L. gasseri* (CST II) and *L. jensenii* (CST V). We also detected a number of significant differences in diversity associated with specific hospitalization reason, again primary in relation to samples with a *L. crispatus* dominance (Fig. 3d). A full accounting of comparisons and significant differences can be found in Supplemental Table S5.

Further examination of the bacterial composition of samples yielded significant differences in the distribution of mothers according to hospitalization reason. We found that individuals admitted for pre-term delivery risk had a VMC dominated by taxa ‘other’ than *L. crispatus, L. iners,* or *G. vaginalis,* compared with admission full-term spontaneous deliveries (chi-squared, p = 7.87 × 10^–4^). Interestingly, we did not identify a significant difference specifically linked to *G. vaginalis* and hospitalization reason*.* Given the significant association with a VMC that appeared potentially dysbiotic, outside of common CSTs, we next looked at reported pregnancy outcomes. Chi-squared testing of samples, binned this time by pregnancy length, showed that a significantly (p = 1.02 × 10^–4^) higher number of samples with shorter pregnancy lengths had VMCs dominated by bacterial species other than *L. crispatus, L. iners*, or *G. vaginalis* (Supplemental Figure S1). The p-value decreased to 2.84 × 10^–8^ when excluding all other *Lactobacillus*-dominated samples. These results, summarized also in Supplemental Table S6, suggest that a combination of non-standard bacterial community combined with a higher overall diversity could be markers of an increased risk of premature delivery.

### Dominant taxa are associated with clinical outcomes

**Figure 4:**
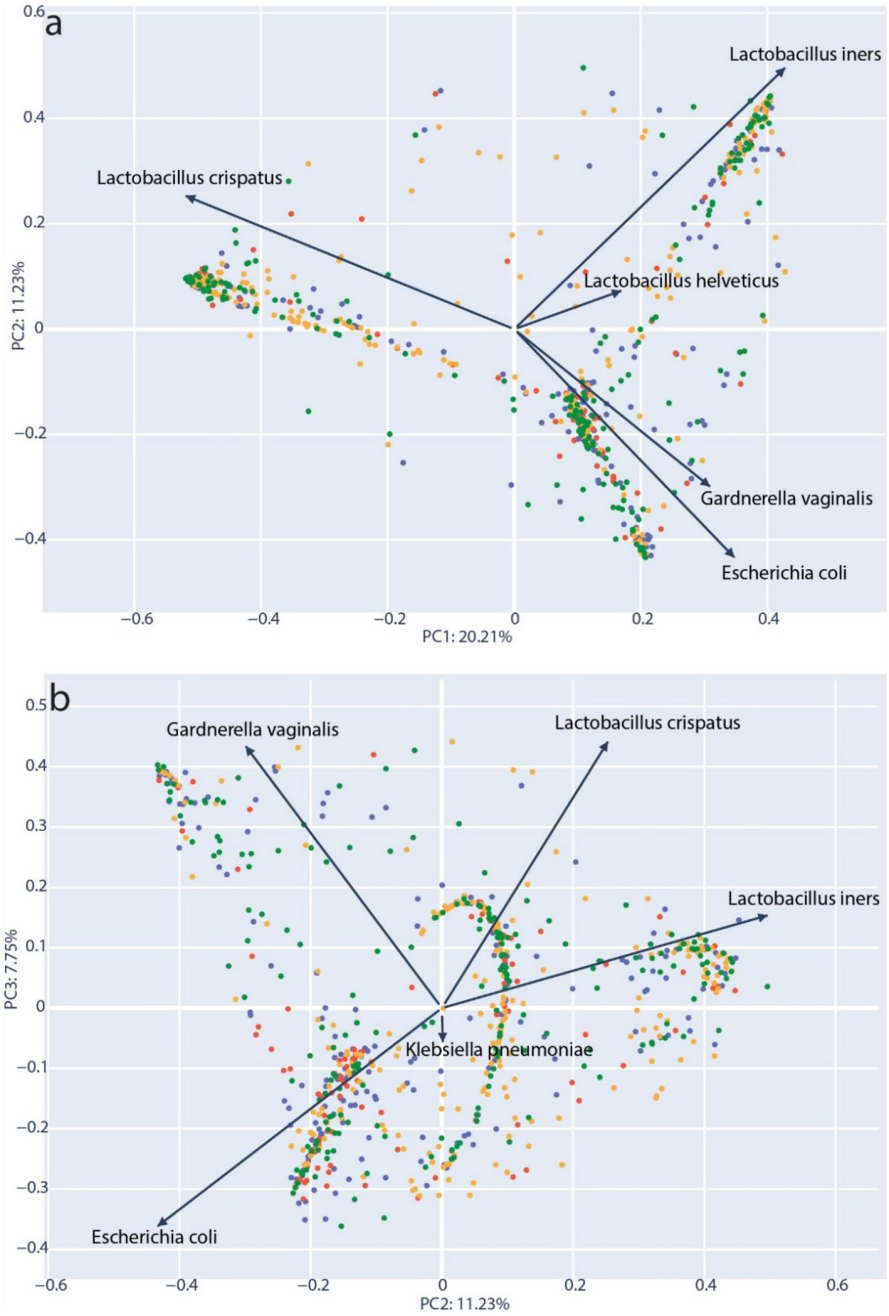
PCoA projection of Bray–Curtis dissimilarity. (**a**) Principal components PC1 and PC2 used for 2D projection for samples. (**b**) PC2 and PC3 used for 2D projection of samples. Individual components (species) that are the primary drivers along each axis are indicated with an arrow representing the strength of each loading proportional to its length. Samples are colored according to their inclusion group: green = control full-term delivery, orange = membrane rupture > 24 h prior to full-term delivery, blue = preterm delivery < 37 weeks, red = preterm delivery with membrane rupture > 24 h prior to delivery.

In order to further define the role of community types and diversity, we performed PCoA analysis^46^ using the normalized taxonomic frequency data from vaginal microbiota samples. Bray–Curtis dissimilarity distances were generated that included all samples and this data projected in two-dimensions. The resulting structure of the PCoA corroborated our observation of VMCs dominated by *L. crispatus*, *L. iners* and *G. vaginalis* (Fig. 4a)*.* We observed that 20.15% of sample variability could be explained by on the primary axis (PC1). The top taxa loading for each axis was plotted as vectors; For PC1, the transition between the two dominant *Lactobacillus* species appeared to be the most important factor separating the samples. Generally, samples could be distinguished by the community abundance of *L. crispatus* versus non-*L. crispatus* species (moving left to right). Figure 4b represents the same data with coordinates plotted for the second and third principal coordinates (PC2 & PC3). Here, two other dominant communities, *Gardnerella vaginalis* and *L. iners*, are clearly separated across the top of the plot and a third community with a strong loading for *E. coli* is placed in the lower half. Interestingly, *Klebsiella*, a genus of opportunistic human pathogens, was observed with a loading similar to *E. coli*, and distanced from the standard CST species. This again suggested that a certain amount of opportunistic pathogen diversity could be associated with divergence from the canonical community types.

We performed PERMANOVA analysis to identify significant groupings based on clinical data for samples in the Bray–Curtis dissimilarity matrix. An analysis of all samples (n = 749) at the species level showed that only the abundance of *Haemophilus influenzae* was significantly associated with the Bray–Curtis groupings (p = 0.038). However, when the analysis was performed by first stratifying samples by their most abundant taxa, we found other significant associations. *L. iners*-dominated samples (n = 160) were found to have significant grouping for both hospitalization reason (p = 0.008) and birth mode (p = 0.043). Within *G. vaginalis*-dominated samples (n = 104), analyzed at the genus level, there were significant associations for frequent urinary tract infections (p = 0.044), umbilical cord inflammation (p = 0.042) and gestation length at time of admittance (p = 0.022). It is notable that *L. crispatus*-dominated samples (n = 251) yielded no significant clinical associations.

### Random forest classifier

Identification of links between microbial diversity and pregnancy length coupled to associations of dominant VMC species to clinical variables of risk, including gestation time and infection, were extremely interesting. We used machine learning as an efficient means to further explore these results through selection and integration of a large number of microbial variables. A Random Forest Classifier (RFC) was trained to integrate the contributions of multiple members of the VCM in order to explain clinical observations. We focused on hospitalization for a risk of premature birth, an important variable in the study.

**Figure 5:**
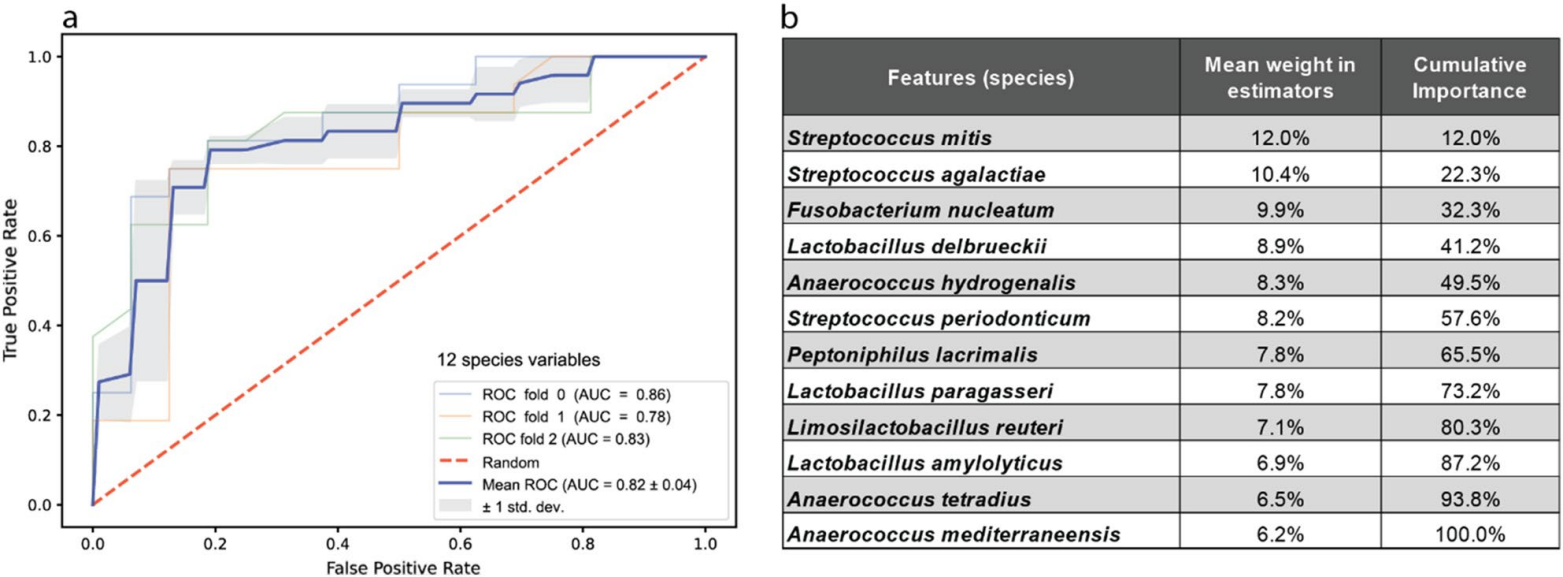
Random Forest predictor of premature birth risk. (**a**) Receiver operator characteristic (ROC) test of the RF classifier for normal and high-risk hospital admissions. Samples are class scored as correctly classified ‘true positives’ or incorrectly predicted ‘false positives.’ Areas under the curve are calculated for three random mixtures of training and test samples, blue, orange, and green lines, compared to an expected random result, dashed red line. (**b**) List of the twelve species components in the best performing RF classifier. The mean weights over individual estimators are given as well as their cumulative weighs in the final predictor.

There was a total of 48 samples from mothers admitted to the hospital due to an elevated risk of premature birth. An equal number of control samples was randomly selected from mothers admitted under the standard procedure for spontaneous labor (Supplemental Table S4). We trained the RFC model on a subset of 133 species variables that were present in at least 25% of samples and which also had a minimum of 50 mean normalized reads across all samples. A preliminary round of training resulted in a classifier that incorporated the frequencies of 109 species variables. The species feature with the highest importance in the 109-species model was *Mageeibacillus indolicus*, a recently isolated bacterium from the female genital tract^47^. Recursive factor removal (RFR) was performed whereby the classifier was refined by eliminating the least important species variable from the data set and retraining with the remaining taxa. This process was run iteratively, testing the accuracy of the resulting classifier at each round. Local regression (LOWESS) applied to a scatterplot of model accuracy indicated that a maximal accuracy was achieved with twelve species variables (Supplemental Table S4). These twelve species were then used to train the definitive classifier. The overall accuracy of the RFC in distinguishing admission for full-term spontaneous births from samples admitted for a risk of premature birth increased from 62.5% percent for the preliminary model with 109 variables to 83.3% with the optimized twelve species. For validation, a receiver operating characteristic (ROC) curve was constructed from three independent combinations of training and testing samples. ROC analysis yielded a mean area under the curve (AUC) value of 0.82 ± 0.04 (Fig. 5a). The twelve most informative species variables (Fig. 5b) are, excepting *Peptoniphilus lacrimalis,* gram-positive with the top three (*Streptococcus mitis*, *Streptococcus agalactiae, Fusobacterium nucleatum*) known as potential pathogens. Notably, the *Lactobacillus* species in the list (*L. delbrueckii, L. paragasseri, L. reuteri, L. anylolyticus*) are not those which normally predominate the major CSTs.

## Discussion

Here we report on a prospective cohort of pregnant women from three Parisian hospitals elucidating the link between the vaginal microbiota and pregnancy outcomes, specifically the risk of preterm delivery. An extensive array of clinical data was collected for both mothers and infants and paired with whole-genome metagenomic sequencing analysis of vaginal swabs collected at the time of delivery. The analyzed composition of the VMC agreed with previous studies as we observed distinct CSTs, primarily dominated by *Lactobacillus* species. In accordance with the majority of previous reports for both child-bearing age women who were not pregnant and those with ongoing pregnancies, we found that *Lactobacillus crispatus* (CST I, 252 out of 749 samples, 33.6% of samples) and *Lactobacillus iners* (CST III, 165 samples, 22.0% of samples) represented the majority of community types. Both *L. gasseri* (CST II) and *L. jensenii* (CST V) were minor CSTs, representing 18 (2.4%) and 21 (2.8%) samples, respectively. As in Brown et al*.,* we observed an increased risk of preterm delivery in mothers with reduced levels of *Lactobacillus*, and a subsequent increase in other bacteria not normally associated with the VCM^48^. We did not, however, identify any significant effect of *L. iners* abundance on pregnancy length or an associated risk of preterm birth^49^. *Gardnerella vaginalis* was found to anchor a third important community state in our cohort (105 samples, 14.0% of samples) most closely related to CST IV. In accordance with a higher diversity associated with CST IV, we noted that *G. vaginalis* accounted for only 62% of reads in samples where it was found to be the most abundant species. This compared to CST I samples where *L. crispatus* accounted for a full 85% of reads and CST III samples where *L. iners* represented 79% of reads. Compared with the *Lactobacillus* species, this result agrees with a number of previous studies reporting an overall higher bacterial diversity, often associated with vaginosis, in samples with higher proportion of *G. vaginalis*^17,50,51^.

Communities with a lower abundance of *Lactobacillus* species lack the exclusionary effect of a low-pH environment normally generated by the production of lactic acid^13,52^. Our own results indicated that *L. crispatus*-dominated samples harbored the lowest overall diversity and that this protective exclusion effect could have important implications in pregnancy. When examining the risk of unfavorable pregnancy outcomes, several reports have pointed to the presence of *Candida albicans* and/or *Ureaplasma* species in preterm labor^31^. We found it interesting to note that co-occurrence of these organisms, which was 21% in the overall cohort, varied significantly depending on the dominant species (chi-squared: p = 1.1 × 10^–4^): 15% for *L. crispatus* dominance, 25% for *L. iners* and 35% for *Gardnerella vaginalis*. While it was not completely unexpected to see that the reduced abundance of major *Lactobacillus* spp. results in a higher colonization rate for *C. albicans, U. urealyticum* and *U. parvum*, the significant differences in co-occurrence in *Lactobacillus*-depleted samples still serves to highlight the protective role of *Lactobacillus* species during pregnancy. An examination of preterm births in our cohort revealed that the level of co-occurrence of *C. albicans* and *Ureaplasma spp.* was 29% for pregnancies lasting < 30 wks.

Our results demonstrating a higher diversity in communities lacking the protective role of *Lactobacillus* combined with the range of species identified in our Random Forest classifier, reinforce the notion that no single species or group of species was solely responsible for an increased risk of preterm delivery. The size and diversity of our cohort allowed us to clearly demonstrate a large number of women with high *Gardnerella vaginalis* abundances who were nonetheless asymptomatic and experienced perfectly normal pregnancies. This observation is particularly relevant for women of African descent who typically display a higher prevalence of *Gardnerella vaginalis*^53^. Our results offer new insights into the complexities in diagnosing vaginal dysbiosis or bacterial vaginosis and assessing phenotypic risk based on the presence of this or any single species^18,54^. It is an intriguing possibility that, in the absence of certain *Lactobacilli*, normally benign species which are usually in low abundance might serve as facilitators to the colonization of pathogens; Gilbert et al*.* reported a significant cooccurrence between *Gardnerella vaginalis* and *Prevotella bivia* in a mouse model*,* with the later species having been previously reported to be linked to *Peptostreptococcus anaerobius* and associated with a higher propensity for pathogenicity^55,56^. Such a scenario could explain the presence of normally rare taxa (*Limosilactobacillus reuteri* and *Peptoniphilus lacrimalis*) as well as the strictly anaerobic *Anaerococcus* species (*A. hydrogenalis*, *A. tetradius* and *A. mediterraneensis*) in our RF classifier (Fig. 5b).

As part of our analyses into microbial diversity in the VMC, we sought to optimally measure both the value and variation of this diversity. Both α- and β-diversity of the VMC is low compared with other body sites, although species-level diversity across distinct CSTs (*Lactobacilli*) can be much higher^45^. In this work we found that Faith’s Phylogenetic Diversity (PD) calculations provided useful insights into the VMC structure and its changes for different *Lactobacillus* species. Compared with alternative measures of α-diversity, such as the Shannon index, often employed in other microbiota studies, including those of the intestinal microbiota, PD integrates taxonomic diversity. This is accomplished by incorporating both phylogenetic distances between individual taxa and their abundances which appears to be more sensitive in identifying biologically significant differences in the VMC. The fact that *L. crispatus* communities were consistently significantly separated from other *Lactobacilli,* as well as *G. vaginalis,* was confirmed in PCoA and was, in turn, linked to clinical consequences such as earlier hospitalization and shorter pregnancy times. Indeed, we found it interesting that PD α-diversity, in comparison to Shannon diversity, appeared to confirm the importance of *L. crispatus* while finding less significant differences between non-*crispatus* communities (Supplemental Table S5). This observation again highlights the role of increased diversity in the VMC over specific CSTs, or specific species.

In conclusion, we find that that a high abundance of *Lactobacillus* spp., particularly *L. crispatus*, suppresses diversity of the vaginal microbiota reducing the risk of colonization by diverse anaerobes and potential pathogens and also reducing the corresponding risk of preterm delivery. Given the protective role that *L. crispatus* plays, future work to examine strain diversity and abundance in relation to human genetic factors would be of high interest. Expression of genes responsible for the production and availability of glycogen (e.g. α-amylase) has been found to be an important factor in determining the VMC composition^9^. Further work should therefore examine pregnancy-related host factors that could be important in promoting the most favorable vaginal microbial communities. Further development of biomarkers that incorporate the quantification of *L. crispatus* strains, as well as a subset of potential pathogens and associated bacterial species holds the promise of better assessing the risks of preterm birth and increasing the survival rate of newborns though better targeted care.

## Supplementary Information

Supplementary Information 1.

Supplementary Information 2.

Consortium members.

## Data Availability

The sequencing data used for analysis in the current study are available in the European Nucleotide Archive (ENA) repository: Project accession PRJEB59811, and files accessions ERS14632248–ERS14632996.
