# Author Correction: Microbial diversity in the vaginal microbiota and its link to pregnancy outcomes

**DOI:** 10.1038/s41598-023-39583-8

**Published:** 2023-08-01

**Authors:** Agnes Baud, Kenzo-Hugo Hillion, Céline Plainvert, Véronique Tessier, Asmaa Tazi, Laurent Mandelbrot, Claire Poyart, Sean P. Kennedy

**Affiliations:** 1 Institut Pasteur, Université Paris Cité, Département de biologie computationnelle, F-75015 Paris, France; 2 AP-HP Centre-Université Paris Cité, FHU PREMA, Centre national de référence des streptocoques, Paris, France; 3 FHU PREMA, AP-HP DRCI, Paris, France; 4 Service de gynécologie-obstétrique, Hôpital Louis Mourier, AP-HP, Université de PARIS, IAME INSERM U1137, Paris, France; 5 Université de Paris, INSERM, Institut Cochin 1016, Paris, France

Correction to: *Scientific Reports* 10.1038/s41598-023-36126-z, published online 04 June 2023

The Acknowledgments section in the original version of this Article did not recognise the InSPIRE Consortium contribution.

“This work was supported by the Programme d'Investissements d'avenir and bpifrance (Structuring R&D Project for Competitiveness—PSPC): # DOS0053477 SUB et DOS0053473 AR.”

now reads:

“This work was supported by the Programme d'Investissements d'avenir and bpifrance (Structuring R&D Project for Competitiveness—PSPC): # DOS0053477 SUB et DOS0053473 AR. A full list of contributors to this study, collectively called the InSPIRE Consortium, can be found in the accompanying Supplementary Information file 3.”

The original Article and its Supplementary files have been corrected.

