# Supplementary material for: Microbial diversity in the vaginal microbiota and its link to pregnancy outcomes
Source: Sci Rep. 2023 Jun 4;13:9061. doi: 10.1038/s41598-023-36126-z (PMC10239749; doi:10.1038/s41598-023-36126-z)

**Supplemental Figure S1 । VCM diversity by dominate species linked to pregnancy length.** (**a**) Faith’s Diversify (PD α-diversity) values for each major dominate community type: *L. crispatus*, *L. iners*, *G. vaginalis*, and all other non-*Lactobacillus* species. Box-and whisker plots are shown for each community for gestations length of 21.7-29.7 weeks (pink), 29.7-33.2 weeks (lime), 33.2-34.4 weeks (yellow), 34.4-36.1 weeks (orange), 36.1-38.2 weeks (green), 38.2-40.0 weeks (light blue). Intervals were chosen so as to obtain approximately equal numbers of samples in each. (**b**) The relative number of samples dominated by a given species: *L. crispatus* (blue), *L. iners* (orange), *G. vaginalis* (grey), and ‘other’ species (yellow). Pregnancy length in weeks, using the same intervals as in ‘a,’ is indicated to the right of each bar.


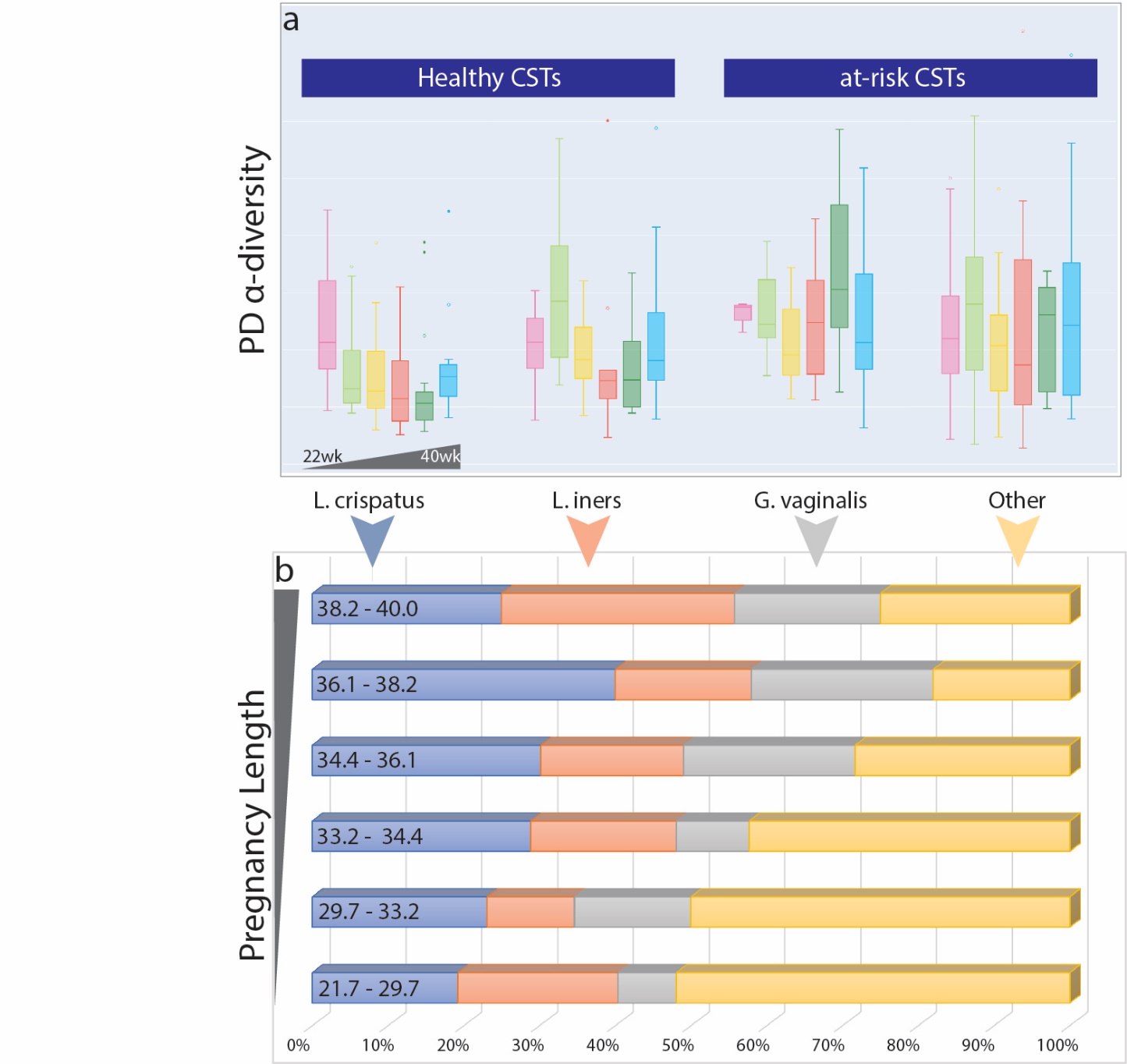

Supplement: Supplementary file 2 — Supplementary Information 2. [file 41598_2023_36126_MOESM2_ESM.docx]
